# Evidence of antagonistic predictive effects of miRNAs in breast cancer cohorts through data-driven networks

**DOI:** 10.1038/s41598-022-08737-5

**Published:** 2022-03-25

**Authors:** Cesare Miglioli, Gaetan Bakalli, Samuel Orso, Mucyo Karemera, Roberto Molinari, Stéphane Guerrier, Nabil Mili

**Affiliations:** 1 University of Geneva, Geneva School of Economics and Management, Geneva, 1205 Switzerland; 2 Auburn University, Department of Mathematics and Statistics, Auburn, AL 36849 USA; 3 University of Geneva, Faculty of Science, Geneva, 1211 Switzerland; 4 University of Lausanne, Lausanne, 1015 Switzerland

**Keywords:** Cancer, Computational biology and bioinformatics, Genetics, Systems biology, Biomarkers, Oncology

## Abstract

Non-coding micro RNAs (miRNAs) dysregulation seems to play an important role in the pathways involved in breast cancer occurrence and progression. In different studies, opposite functions may be assigned to the same miRNA, either promoting the disease or protecting from it. Our research tackles the following issues: (i) why aren’t there any concordant findings in many research studies regarding the role of miRNAs in the progression of breast cancer? (ii) could a miRNA have either an activating effect or an inhibiting one in cancer progression according to the other miRNAs with which it interacts? For this purpose, we analyse the AHUS dataset made available on the ArrayExpress platform by Haakensen et al. The breast tissue specimens were collected over 7 years between 2003 and 2009. miRNA-expression profiling was obtained for 55 invasive carcinomas and 70 normal breast tissue samples. Our statistical analysis is based on a recently developed model and feature selection technique which, instead of selecting a single model (i.e. a unique combination of miRNAs), delivers a set of models with equivalent predictive capabilities that allows to interpret and visualize the interaction of these features. As a result, we discover a set of 112 indistinguishable models (in a predictive sense) each with 4 or 5 miRNAs. Within this set, by comparing the model coefficients, we are able to identify three classes of miRNA: (i) oncogenic miRNAs; (ii) protective miRNAs; (iii) undefined miRNAs which can play both an oncogenic and a protective role according to the network with which they interact. These results shed new light on the biological action of miRNAs in breast cancer and may contribute to explain why, in some cases, different studies attribute opposite functions to the same miRNA.

## Introduction

Breast cancer (BC) is the second-most common cancer and second-leading cause of cancer mortality in American women. In the USA, its incidence in 2019 was roughly 268,600, and it is responsible for an estimated 41,760 deaths^1^ to which one must add 62,930 new cases of Ductal Carcinoma In Situ (DCIS). In Norway, where the data analysed in this work were collected, breast cancer comprises more than 22% of all cancer cases in women, and the current incidence indicates that one in twelve women will be diagnosed with breast cancer by the age of 75^2^. In 2018 breast cancer was the most common female cancer in the European Union, accounting for 29.2% of all cancers in women. A total of 404,920 new female breast cancer cases was estimated to have occurred in 2018, corresponding to an age-adjusted standardized rate of 144.9/100,000^3^.Breast cancer is therefore a key public health issue in Europe and in the USA (as well as in many other regions).

Dysregulation of microRNAs (miRNAs) plays a key role in almost all cancers, including BC^4^. miRNAs are short endogenous noncoding RNAs that regulate their target messenger RNAs (mRNA) by promoting mRNA degradation or repressing translation. Chang et al.^4^ found that increased expression of 12 mature miRNAs—hsa-miR-320a, hsa-miR-361-5p, hsa-miR-103a-3p, hsa-miR-21-5p, hsa-miR-374b-5p, hsa-miR-140-3p, hsa-miR-25-3p, hsa-miR- 651-5p, hsa-miR-200c-3p, hsa-miR-30a-5p, hsa-miR-30c-5p, and hsa-let-7i-5p—all predicted improved BC survival. In a recent review, Adhami et al.^5^ determined that two miRNAs (hsa-miR-21 and hsa-miR-210) were upregulated consistently and six miRNAs (hsa-miR-145, hsa-miR-139-5p, hsa-miR-195, hsa-miR-99a, hsa-miR-497 and hsa-miR-205) were downregulated in at least three studies. In another study, Haakensen et al.^6^ identified some miRNA alterations during BC progression. These alterations were involved in the invasive signatures of BC including downregulation of hsa-miR-139-5p in aggressive subtypes and upregulation of hsa-miR-29c-5p in luminal subtypes. A total of 27 miRNAs were implicated in their proposed DCIS signature.

The latter study provided one of the main reasons to develop the work presented here. Indeed, Haakensen et al.^6^ provide the following statement in their article: “*hsa-miRNA-210-3p was significantly upregulated in both our analyses, but was downregulated in the same transition in Volinia et al.*^7^ *and is hence excluded from our proposed signature*”. Following on this statement, hsa-miR-210 had previously been identified as a marker of poor prognosis in BC and other carcinomas^8^. In fact, Volinia et al.^7^ found hsa-miR-210 to be downregulated in DCIS compared to normal breast tissue, but upregulated in invasive carcinomas compared to DCIS. In addition, Shao et al.^9^ recently showed that hsa-miR-210 is associated with internal organ metastasis (liver, lung, and brain) in BC. In Haakensen’s study, hsa-miR-210 was upregulated in DCIS compared to normal tissue and was not detected as significantly altered in any invasive subtype. Given the unclear role of this miRNA in breast carcinogenesis, Haakensen’s study therefore discarded it from the list of miRNAs involved in BC progression.

Considering these studies, this work aims at addressing some questions that naturally arise from their conclusions. The first of these questions is as follows: why weren’t there any concordant findings in many research studies regarding the role of miRNAs in the progression of BC? Are the different outcomes due to population selection, batch effect or to biological causes such as disease heterogeneity, overlapping of miRNA functions or network effects? A second question, that stems from the latter points, is the following: could a miRNA have either an activating effect or an inhibiting one in a given biological process (such as cancer progression) according to the other miRNAs with which it interacts? In other words, could a specific miRNA be upregulated in one study and downregulated in another as a result of the complex pathways in which it is involved (instead of this being the effect of the experimental conditions)? This work aims at investigating these questions more thoroughly and, inspired from the work of Stepanenko et al.^10^, we will refer to miRNAs with such contrasting effects as *antagonistic*. However this definition has no mechanistic causal claim within the framework of this work since such a notion would have to be further investigated by experimental validation. With this in mind, to minimize the impact of factors such as population selection^11^, batch effect^12^ and experimental conditions (e.g. the specific machine that extracts the features^13^), we decide to focus on a single set of data where these effects can be considered reasonably constant. In particular, we analyze the AHUS (Akershus University Hospital) dataset using a recently proposed algorithm, called SWAG^14^ (the acronym of “Sparse Wrapper AlGorithm”). The dataset is made available by Haakensen et al. on the open access ArrayExpress platform at: https://www.ebi.ac.uk/arrayexpress/experiments/E-MTAB-3759/?query=AHUS. To promote reproducibility and replicability, we make the SWAG available as an R package on CRAN and at https://github.com/SMAC-Group/SWAG-R-Package/ for its development version. We employ this algorithm to build the set of highly predictive genomic models presented in the results section.

Our work aims at contributing to the field of systems biology^15^ where the use of mathematical and computational models applied to biology is of the uttermost importance. Systems biology is a field which, among others, focuses on the assumption that a discrete biological function can rarely be attributed to a single molecule^16^. Instead, most biological characteristics arise from complex interactions among the cell’s numerous constituents, such as proteins, DNA, RNA and small molecules. Understanding the structure and the dynamics of complex intercellular networks that contribute to the structure and function of a living cell is therefore paramount before assigning a function to any biological feature^17^. According to Barabási et al.^18^, the inter- and intra-cellular connectivity implies that the impact of a specific genetic abnormality is not restricted to the activity of the gene product that carries it, but can spread along the links of the network and alter the activity of gene products that otherwise carry no defects. However, the biological networks in which a single genomic variable is involved remain unknown and as a first step, one should then rely on a data-driven network built using statistical (and not biological) associations. In summary, our study has three goals: (i) to investigate if, and to what level of accuracy, it is possible to use different combinations of miRNAs as biomarkers to discriminate normal breast tissue from breast carcinoma; (ii) to check how the behaviour of these miRNAs varies according to the specific combination with which they interact; (iii) to search for interchangeable miRNAs in these predictive models and by doing so, to decipher the biological targets of these variables.

## Methods

### Genomic study

The results of our research are based on the AHUS dataset made available on the ArrayExpress platform by Haakensen et al.^6^. According to the authors, in order to collect this data the Akershus University Hospital sequentially collected breast tissue specimens from BC patients and from women undergoing surgery for breast reduction. These specimens were collected over 7 years between 2003 and 2009. miRNA-expression profiling was obtained for 55 invasive carcinomas and 70 normal breast tissue samples (including 29 tumor-adjacent normal tissue samples and 41 breast reduction samples) for a total of 125 as stated on the ArrayExpress platform. The samples were hybridized on Agilent 8x15K arrays (Agilent Technologies, Santa Clara, CA), catalogue number 4470B (v2) and 4470C (v3), and the features were extracted using Agilent Feature Extraction. Relevant information can be found in Haakensen et al.^6^.

### Statistical analysis

When considering the research goals defined earlier, the statistical tools used to achieve them need to be defined accordingly. Hence, the first step is to find “different combinations of miRNAs” which implies that we are not aiming to find a single statistical (or machine learning) model to classify normal breast tissue and breast carcinoma. Indeed, we intend to find a variety of models (miRNA combinations) that all perform this classification task with a high level of accuracy and renders them equivalent in terms of predictive power. The idea of considering a multitude of models is not a common one but has been put forward in different settings (see e.g. Caruana et al.^19^) and was adequately stressed, for example, in Whittingham et al.^20^ who state that “[...] *further analysis should not be based on a single best model, but should explicitly acknowledge uncertainty among models that are similarly consistent with the data*”. In fact, depending on the setting, the reliance on a single model can be rather risky and can often deliver contradicting results regarding if and how certain variables contribute to explain or predict a given phenomenon of interest. In this perspective, we should choose an approach that allows to find a variety of “strong” models and that, in accordance with the subsequent research goals of this work, can be used to create miRNA networks highlighting how, for example, a specific miRNA can be used to detect (and can contribute differently to) breast carcinoma when considered with other miRNA combinations. In addition, in order to create networks that can be interpreted from a biological perspective, we need these models also to be based on small (sparse) combinations of miRNAs.

There exist a wide variety of statistical and machine learning approaches to select and estimate models with few features (miRNAs) but, in most cases, these only select one model which therefore limits the possibility of considering how the impact of an miRNA can change when considered with another set of variables. For this reason this work uses the “Sparse Wrapper AlGorithm”(*SWAG*) put forward in Molinari et al.^14^ which is described in the following paragraphs.

#### A wrapper method for sparse learning

The *SWAG* is a method derived from the Panning algorithm presented in Guerrier et al.^21^ for gene selection problems. The premise of this method is the assumption that, in order to adequately predict a certain outcome of interest (e.g. breast carcinoma), we only need an extremely small set of features and that there are many models (combinations of small sets of features) that can all have equivalent and high predictive power. Aside from allowing to understand if and how certain features can behave differently when considered in presence with other sets of features, the output of the *SWAG* also allows to facilitate replicability of results. Indeed, when a study proposes a single model (and hence a single combination of features) in order to detect or predict a certain response, this may not be usable for a research or medical structure that may not have the possibility of measuring all the selected features.

**Figure 1:**
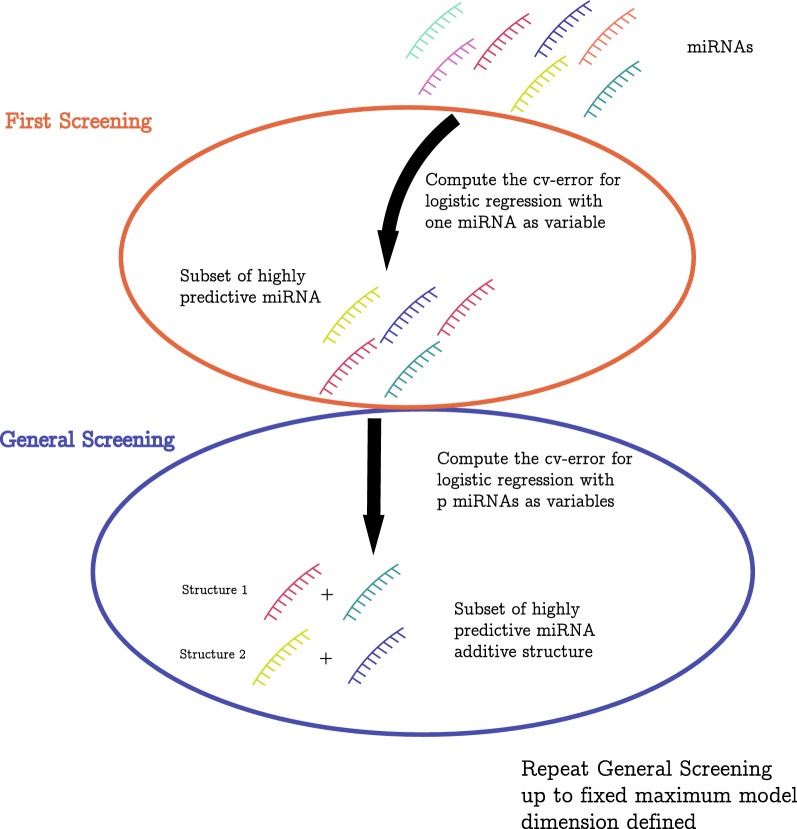
*SWAG* flowchart. A schematic representation of how the *SWAG* was calibrated for this work. The red set represents the first step which evaluates every one-dimensional model and selects the best expressions to be used in the general step represented by the blue set. The latter step evaluates and selects the best models of dimension 2 to $p_{\text {max}}$.

In order to respond to the above needs, the *SWAG* consists in a “greedy” wrapper algorithm that firstly requires the user to specify a model (or learning method), such as a logistic regression model, as well as the maximum number of variables ($p_{max}$) to be considered within such a model. The latter choice can be made, for example, based on prior knowledge of the problem and interpretability requirements (the smaller this number, the easier the output will be interpreted). Based on these choices and supposing there is a total of *p* features (e.g. biomarkers), the *SWAG* starts through a first screening step where *p* models are built, each using a distinct feature. At this stage, the out-of-sample prediction error of each model can be estimated via *k*-fold cross-validation repeated *m* times and the best of these models (in terms of lowest prediction error) can be selected thereby providing a list of features that, on their own, appear to be highly predictive for the considered response. The definition of “best” models will be given by the user through a parameter $\alpha$ which represents a proportion (or percentile) and is usually chosen to be considerably small (i.e. between 0.01 and 0.1). With smaller values of $\alpha$ implying a more strict selection of best models (hence the choice of only the most performing features), the *SWAG* then uses the features selected in the first step to progressively build higher-dimensional models (i.e. models with an increased number of feature combinations within them) until it reaches the maximum number $p_{max}$. When building the models for a given dimension, the *SWAG* takes the best models from the previous step (i.e. the step that built models with one less feature than the current step) and randomly adds a distinct feature from the set of features selected at the first step. Having built *m* models at each step (where *m* is also chosen by the user), the final output of the *SWAG* is a set of “strong” models (i.e. models with high predictive power) where each is based on a combination of 1 to $p_{max}$ features. A simplified representation of the *SWAG* is presented in Fig. 1. With this output, it is then possible for the user to apply post-processing to select a subset of interest from this set of models.

#### Software information

The *SWAG* is made available as an R^22^ package on the CRAN repository. At the same time, a development version can also be found at https://github.com/SMAC-Group/SWAG-R-Package/. All analyses and figures presented in this paper have been done on R^22^ (version 3.6.0) except Fig. 1 which has been generated with Adobe Illustrator (https://adobe.com/products/illustrator) version 2020 (24.1) for descriptive purposes.

#### Implementation

The AHUS dataset is split into training and test subsets. The training subset contains 100 observations with a 56/44 split (normal tissue/invasive BC). The test subset has 25 observations with a 14/11 split. The *SWAG* learns only on the training data the set of highly predictive models (i.e. combination of miRNAs). These models have a low prediction error (known as counting or classification error in the logistic regression case) because the *SWAG* at each step selects the features (i.e. miRNAs) with the smallest 10-fold cross-validation (repeated 10 times) error where we have fixed the value of the constants *k* and *m* to the standard value of 10. Indeed with cross-validation, we estimate how accurately a predictive model will perform in practice. This is a well-established model validation technique for assessing how the results of a statistical analysis will generalize to an independent dataset. The aim is to estimate how accurately a model will perform in terms of prediction and the rationale of using this technique may be found in Fushiki^23^ and Molinaro^24^. The caveats of cross-validation are well explained in Bernau et al.^25^ where the main setback can eventually consist in an overestimation of model performance in a broader application context. To avoid this issue, we present in the Results section only the prediction errors of the models obtained in the test data as it is usually done in the machine learning community. In the logistic regression case this implies simply to count how many times the model predicts correctly a unit it has never seen (i.e. because it belongs to the test set) and divide this number by the total number of units predicted (25 in our case).

However our research question is not about the validity of a given model selection method, but about the function of a specific miRNA in BC oncogenesis. To address this issue, we must not only select a set of models in which this specific miRNA is involved, but also determine the direction in which it acts (oncogenic or protective effect). Prior to this analysis we standardized (i.e. centered and rescaled) the design matrix (miRNAs) to ensure a meaningful comparison across the different models. Then, to assess the role of the relevant miRNAs selected via the *SWAG*, we computed their $\beta$ coefficients by performing a logistic regression (see e.g. chap. 6 in Vittinghoff et al.^26^ for a detailed overview) on each element of the set of models. The evaluation of the $\beta$ coefficients allows us to (i) identify either the oncogenic or protective effect of the variable and (ii) gain insight on its distribution. Positive values of $\beta$ mean that the miRNA associated with this coefficient has an activating effect on tumor progression (oncogenic effect); a negative value means the opposite (protective effect). Since the miRNAs can be included in different models delivered by the *SWAG*, one can compute an empirical distribution of the coefficients.

#### Single and associative effects on a binary variable

One of our research questions is whether a given miRNA has the same action (oncogenic or protective) when it is taken in isolation or when embedded within different models and feature combinations. In order to assess the biological action of the selected miRNAs, we compute both the single and associative effects of the $\beta$ coefficients for each of the selected miRNAs. A single effect is measured by the estimated value of a $\beta$ coefficient when considering a single miRNA in the logistic model. The associative effect is defined as all the different values (i.e. range) that a $\beta$ coefficient takes within the set of models, discovered by the *SWAG*, which contain that given miRNA. The associative effect of a specific miRNA therefore may be seen as an indicator of its biological impact in a broader context.

As a matter of fact, according to Cox^27^, these effects are typically different. For a random variable considered both alone and conditionally on a confounding variable W, the single and associative inferences may have opposite signs by the so-called Yule-Simpson effect^28^. This effect, if observed, may be explained in two ways: (i) the existence of subpopulations or (ii) the influence of a finite set of latent classes W (such as biological functions) within the population under study. An example of this phenomenon with some mathematical explanations can be found in Cox^27^ and Boehm et al.^29^. Splitting the population into defined subgroups may, to some extent, dodge the first pitfall (i.e. the existence of subpopulations). However, BC heterogeneity is large and has been documented in terms of different histological subtypes, treatment sensitivity profiles, and clinical outcomes. Furthermore, the heterogeneous expression of the oestrogen receptor, progesterone receptor, and HER2 has been reported in different areas of the same tumour. Molecular profiling studies have confirmed that spatial and temporal intratumour heterogeneity of BCs exist at a level beyond common expectations^30^. Splitting BC populations into subtypes may then be a misleading precaution. The second pitfall (i.e. the existence of latent biological functions shared by many genomic features) is even more elusive. Having not a single, but a set of predictive models may help get around this hurdle. We addressed this challenge (differentiating the effect of subpopulations from that of latent biological variables on single and associative coefficients) by mixing the 55 invasive carcinomas into one category. If single and associative coefficients retain the same sign throughout the 112 selected models, we can conclude with some confidence that the existence of subpopulations (BC sub-types) has no effect on the oncogenic or protective effect of the relevant miRNAs inside the AHUS dataset. On the contrary, if single and associative coefficients have opposite signs, then one can assume that the effect of the relevant miRNA differs according to its environment.

### Horizontal and vertical organizations

We make the heuristic hypothesis that the human genome as a whole and its sub-units (such as non-coding RNAs) can be interpreted as semiotic systems. To give meaning to the miRNA-based net-like structures that we build through our statistical analysis, we borrow the notions of syntagm and paradigm from structural semiotic analysis, inspired by de Saussure theory^31^. A simple and useful introduction to semiotics may be found in Chandler^32^. De Saussure emphasized that meaning (in our case, oncogenic or protective effect) arises from differences between signifiers (in our case, miRNAs). These differences are of two kinds: syntagmatic (concerning positioning within a model) and paradigmatic (concerning substitution within a given model). These two dimensions are often presented as axes, where the horizontal axis is the syntagmatic and the vertical axis is the paradigmatic. The plane of the syntagm is that of the combination of signifiers (i.e. selected miRNAs) within a statistical model, while the plane of the paradigm is that of the selection of signifiers. Whilst syntagmatic relations are combination possibilities, paradigmatic relations are functional contrasts. The meaning of a signifier is determined by both its paradigmatic and syntagmatic relations. According to this conception, the set made of all the selected models may be seen as the set of syntagmatic ”sentences” selected by the *SWAG*, and the set made of the selected miRNAs as the set of paradigmatic Omics features. In this study, the horizontal syntagmatic axis was used to tackle the second research question (to check how the behaviour of the miRNAs varies according to the specific combination with which they interact). The vertical paradigmatic axis was used to address the third research question (to search for interchangeable miRNAs in these predictive models and, by doing so, to decipher the biological targets of these miRNAs).

## Results

### Breast cancer/normal tissue discrimination

**Figure 2:**
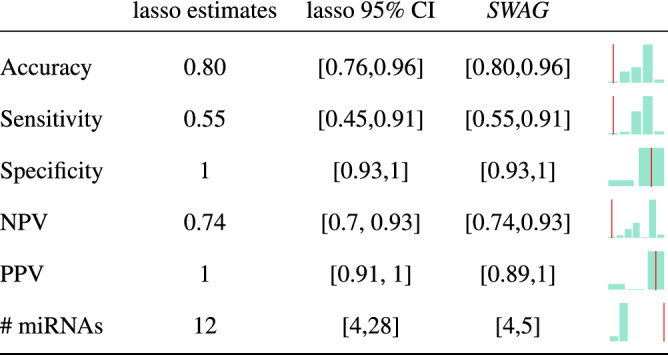
Comparison between Lasso and *SWAG*. We compare accuracy, sensitivity, specificity, negative predictive value (NPV), positive predictive value (PPV), number (#) of miRNAs of the lasso estimates (and relative 95% percentile bootstrap confidence intervals) with the ranges (i.e. smallest-to-largest value intervals) of the same metrics for the 112 *SWAG* models. On the right of each *SWAG* range, a barplot illustrates the distribution of the specific metrics for the 112 considered models with a vertical red line representing the corresponding value for the lasso. All evaluations have been made out-of-sample (i.e. on the test set) at the standard 0.5 cut-off of logistic regression.

**Figure 3:**
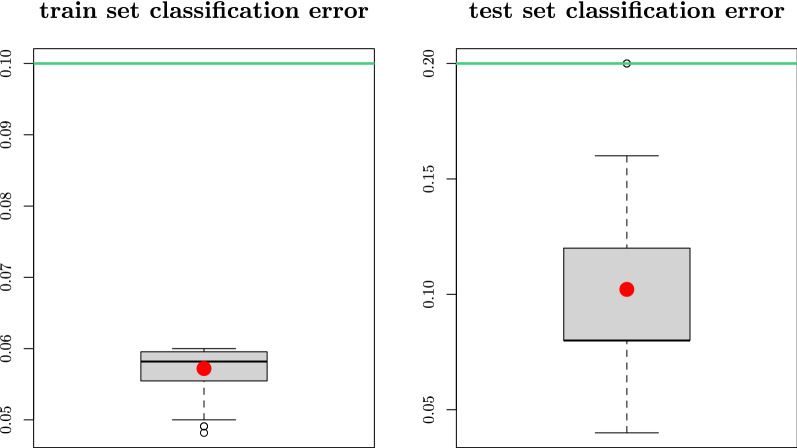
Train and test set classification error for *SWAG*. We compare the box plots of the train set and the test set cross-validation prediction error (i.e. classification error) for the 112 *SWAG* models. The red point inside each box plot represents the average classification error of the *SWAG* models either in the train set or in the test set. The horizontal green line in both plots, that we use as a reference level, represents the classification error of the lasso either in the train or in the test set.

**Figure 4:**
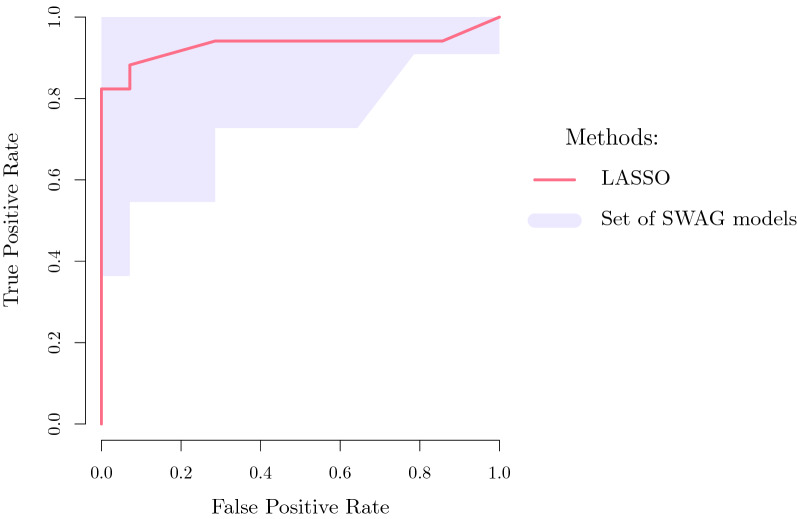
ROC curve comparison between lasso and *SWAG*. We present the ROC curve of lasso (in red) with the ROC region (in gray) produced by the 112 *SWAG* models. We obtain the ROC region for the set of *SWAG* models considering first all the 112 individual model ROC curves and then coloring in gray the area which encloses all the 112 ROC curves at the same time.

When applying *SWAG* to the AHUS data, a total of 45 miRNAs were selected, making a set of 112 indistinguishable models (in a predictive sense) each with 4 to 5 miRNAs. Both the 45 selected miRNAs (see S1 Table 1) and the 112 models (see S1 Tables 2, 3, 4, 5) are presented in the supplementary information material. They perform similarly or outperform the lasso, a standard model selection method used in genomics^33^, with less than half the number of miRNAs selected by the latter. This is evident from the comparison in terms of accuracy, sensitivity, specificity as well as positive and negative predictive values (see e.g. Parikh et al.^34^for a detailed explanation of these metrics) at the standard logistic cut-off level of 0.5 (cf. Fig. 2). Indeed all the 112 *SWAG* models have an equal or greater out-of-sample (i.e. on the test set) accuracy than the lasso estimates, while we see that the lasso 95% confidence intervals for the considered metrics are comparable to the *SWAG* set. It is worth noting that the *SWAG* set achieves such a performance with models of smaller size than the lasso, hence easing the interpretability of the outputs. When looking at the lasso estimates, we see that a large majority of the *SWAG* models (i.e. 97 out of 112) perform better than the lasso in all of the considered metrics. This can be inferred also visually in Fig. 2 by looking at the barplots on the right of each specific *SWAG* range (i.e. the interval between the smallest and largest values among all *SWAG* models), with the vertical red line representing the corresponding lasso performance. To support these findings, we also present (cf. Fig. 3) the box plots of the training and test set classification errors (i.e. the cross-validation prediction errors) for all the *SWAG* models. The green horizontal line in each box plot represents the classification error of the lasso which we use as a reference level. We can notice that every model in the *SWAG* set has a smaller or equal classification error compared to the lasso both in the training set and in the test set. In addition, to allow the comparison also considering different cut-off levels, we present (cf. Fig. 4) the ROC curve of lasso (in red) with the ROC region (in gray) produced by the 112 *SWAG* models. We obtain the ROC region for the set of *SWAG* models considering all the 112 individual model ROC curves and then filling the area which encloses all the 112 ROC curves jointly. Moreover, we can compare the performance of the methods also through the lens of the Event Per Variable (EPV) metric (see e.g.^35–37^). A recent study^37^ links this metric to the out-of-sample performance of a given model (the higher the EPV, the better the external validity). In logistic regression the number of events correspond to the size of the smallest of the outcome categories (i.e. the number of invasive BC for the AHUS dataset). Due to the limited number of miRNAs in each *SWAG* model, we reach an EPV of 8.8 for the models of size 5 and an EPV of 11 for the models of size 4. These values are commonly considered safe^36^ while an EPV smaller than 4, such as the one (i.e. 3.67) reached by lasso, is more problematic. Thus, as a whole, these results suggest that *SWAG* more precisely targets the set of miRNAs involved in BC progression. Furthermore, Table 1 reports two specific *SWAG* models, among the 112 selected ones, that achieve a perfect out-of-sample classification in terms of area under the curve (AUC). It is therefore possible to discriminate BC from normal breast tissue with extreme accuracy using miRNAs as biomarkers. The added value of the *SWAG* compared to lasso is that (i) it produces a set of equivalent models instead of a single one and (ii) the number of selected variables per model is smaller by a factor of two, making the models more easily interpretable.

**Table 1:** Best out-of-sample *SWAG* models that achieve a perfect classification in terms of area under the curve (i.e. $AUC = 1$).

	Non-coding miRNAs
Model 1	hsa-miR-1274a hsa-miR-21 **hsa-miR-92a** hsa-miR-328 hsa-miR-140-3p
Model 2	hsa-miR-1274a hsa-miR-21 **hsa-miR-92a** hsa-miR-328 **hsa-miR-30b**

The non-coding miRNAs are displayed in order of presence in the *SWAG* chain.

The *antagonistic* miRNAs are presented in bold.

**Figure 5:**
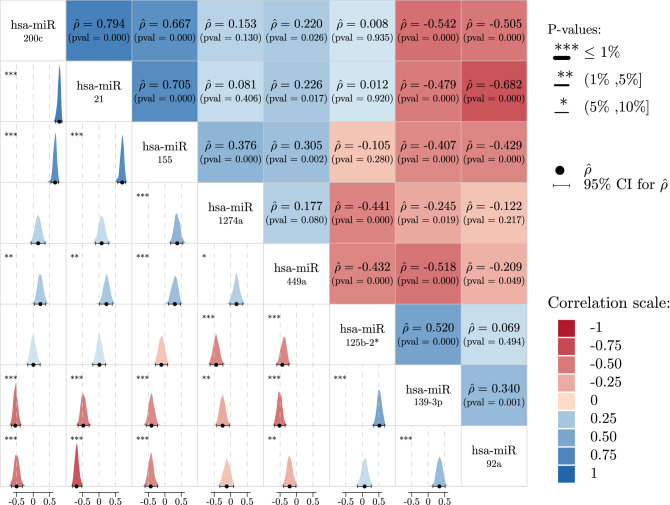
Spearman correlation ($\hat{\rho }$) matrix for the 8 most frequent miRNAs selected by the *SWAG* The upper triangular part shows the estimator $\hat{\rho }$ between miRNAs and their respective p-value computed via non-parametric bootstrap. The color of the boxes indicates the direction of $\hat{\rho }$ (blue for positive correlation and red for negative). The lower triangular part illustrates the bootstrap distribution of $\hat{\rho }$ via the density plot, with the dark circle being the estimator of $\hat{\rho }$ and the horizontal black line its 95% confidence interval. The star on the boxes’ upper-left indicates the level for which the correlation in significant.

**Figure 6:**
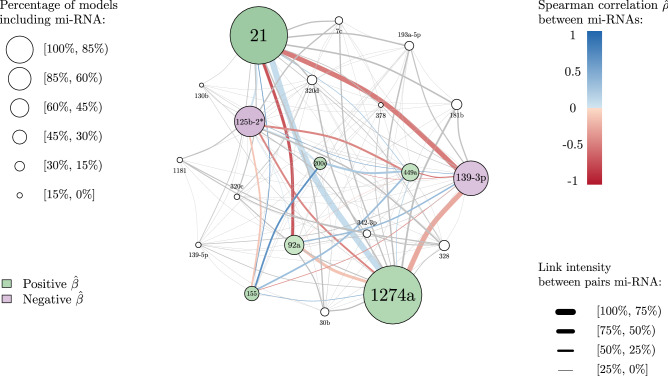
*SWAG* network of the AHUS dataset. Each node represents an miRNA which appears in a given model at least once. The colour of the node reflects the sign of the median of the estimated $\beta$ coefficients (i.e. the median of the different values that a miRNA specific $\beta$ coefficient takes in each of the *SWAG* models in which it is present). The size of each node is proportional to the percentage of models that contain that specific miRNA among all the 112 *SWAG* models. The thickness of each link between different nodes (i.e. miRNAs) is proportional to the percentage of times the two miRNAs appear together among all the 112 models. The colour of the link reflects the value of the estimated Spearman correlation coefficient $\hat{\rho }$ between two different miRNAs (blue for positive correlation and red for negative).

Among the 45 selected miRNAs, 8 were present in more than 16 % of all *SWAG* models. These 8 miRNAs are displayed in Table 2, with their respective pairwise Spearman correlations, illustrated in Fig. 5. As a non-parametric measure of rank correlation, Spearman correlation assesses how well the relationship between two variables can be described using a monotonic function. It is used in our study as an index of result consistency. Two miRNAs having similar effects on cancer progression should be positively correlated. The rational of this statement is that correlation and mutual information are closely related^38^. Selected miRNAs are endowed with both *single* and *associative* $\beta$ coefficients, which are in most cases either overall positive (oncogenic effect) or overall negative (protective effect on tumour progression). We recall that a *single* effect of a given miRNA is measured by the estimated value of a $\beta$ coefficient when considering only that single miRNA in the logistic model. On the other hand, the *associative* effect is defined as all the different values (i.e. range) that a $\beta$ coefficient takes within the set of models, discovered by the *SWAG*, which contain that given miRNA. We have discussed in detail the statistical aspects of this distinction in the Methods section. Based on this approach, we are able to identify hsa-miR-92a as a possible *antagonistic* miRNA since its associative $\beta$ is always positive while the single one is negative (cf. Table 3). This point will be discussed later. To conclude this discussion, we can visualize all these findings with the *SWAG* network for the AHUS data set shown in Fig. 6, allowing for an intuitive interpretation of these results.

**Table 2:** Model occurrence rate of the most frequently selected miRNAs.

Non-coding miRNA	Model occurrence rate (%)
hsa-miR-1274a	75.0
hsa-miR-21	74.1
hsa-miR-139-3p	44.6
hsa-miR-125b-2*	39.3
hsa-miR-92a	25.0
hsa-miR-449a	22.3
hsa-miR-155	18.8
hsa-miR-200c	16.1

**Table 3:** Single (i.e. the estimated value of a $\beta$ coefficient when considering a single miRNA in the logistic model) and associative (i.e. the different values that a miRNA specific $\beta$ coefficient takes in each of the *SWAG* models in which it is present) coefficients (median values and range) for the eight most frequently selected miRNAs.

miRNA	Single $\beta$	Median associative $\beta$	Associative $\beta$ Range
hsa miR-1274a	1.427	2.120	(0.768; 3.392)
hsa-miR-21	1.996	3.174	(1.858; 4.880)
hsa miR-139-3p	− 2.191	− 0.979	(− 1.799, − 0.443)
hsa-miR-125b-2*	− 1.106	− 1.510	(− 2.451; − 1.003)
hsa-miR-92a	− 0.736	0.939	(0.095; 1.315)
hsa-miR-449a	3.672	1.228	(0.379; 2.644)
hsa-miR-155	2.973	1.806	(0.294; 1.920)
hsa miR-200c	1.628	1.600	(0.907; 2.153)

### Oncogenic or protective role of miRNAs: the syntagmatic axis

**Figure 7:**
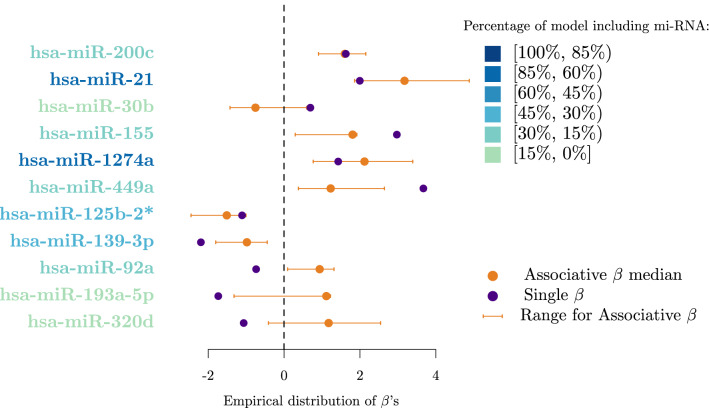
Distribution of $\beta$ coefficients for the most frequently selected miRNAs. We present the single effect (i.e. the estimated value of a $\beta$ coefficient when considering a single miRNA in the logistic model), the median and range of the associative effect (i.e. the different values that a miRNA specific $\beta$ coefficient takes in each of the *SWAG* models in which it is present) for each of the most frequently selected miRNAs displayed in both Tables 3 and 4.

The values of the single and associative coefficients of the eight most frequently selected miRNAs are displayed in Fig. 7 and Table 3. Coefficients related to miRNAs that are present in at least 10% of models and that show discordant values between single and associative $\beta$s are presented in Table 4. Among the eight most frequently selected miRNAs, the median value of the $\beta$s considered along with their range makes it possible to identify three classes of miRNA (cf. Fig. 7 and Table 3): (i) oncogenic miRNAs with single and associative positive values of $\beta$ (hsa-miR-1274a, hsa-miR-21, hsa-miR-449a, hsa-miR-155, hsa-miR-200c); (ii) protective miRNAs (hsa-miR-139-3p, hsa-miR-125b-2*); (iii) an undefined miRNA (hsa miR-92a) with a negative single coefficient and a positive associative one. An indication of the consistency of these results lies in the Spearman correlation coefficients $\hat{\rho }$ (cf. Fig. 5): hsa-miR-1274a is significantly positively correlated (at the level $\alpha = 5\%$) to its oncogenic partner hsa-miR-155 and negatively correlated to protective miRNAs hsa-miR-125b-2* and hsa-miR-139-3p. The same consistency can be observed for hsa-miR-21. The two protective miRNAs, hsa-miR-139-3p and hsa-miR-125b-2*, are also significantly positively correlated with each other, and significantly negatively correlated to oncogenic miRNAs such as hsa-miR-449a and hsa-miR-1274a.

**Figure 8:**
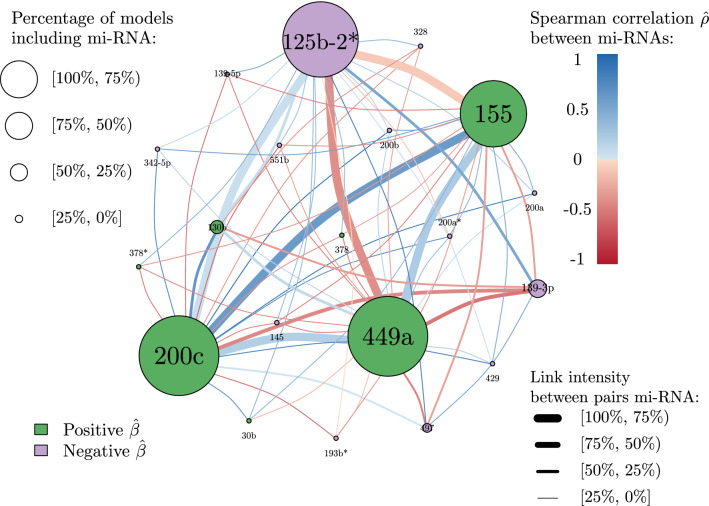
Network of hsa-mir-200c. Each node represents a miRNA which appears coupled with hsa-mir-200c in a given model at least one time. The colour of the node reflects the sign of the estimated $\beta$ coefficient of a logistic regression considering that specific miRNA alone (i.e. single effect). The size of each node is proportional to the percentage of models that contain that specific miRNA among all the models that contain hsa-mir-200c. The thickness of each link between different nodes (i.e. miRNAs) is proportional to the percentage of times the two miRNAs appear together among all the models which contain hsa-mir-200c. The colour of the link reflects the value of the estimated Spearman correlation coefficient $\hat{\rho }$ between two different miRNAs (blue for positive correlation and red for negative).

The case of hsa-miR-92a that displays discordant single and associative coefficients is not isolated. Among the miRNAs selected in at least 10% of the models, three show a similar behaviour to that of hsa-miR-92a: hsa-miR-320d, hsa-miR-193a-5p and hsa-miR-30b. Using a bioinformatics-based interaction analysis of hsa-miR-92a-3p and key genes in tamoxifen-resistant BC cells, Cun et al.^39^ found that hsa-miR-92a-3p was higher in BC serum or tissue than in healthy volunteer serum or adjacent normal tissue. Hence, a high expression of hsa-miR-92a-3p seems to predict poor prognosis for BC patients according to this meta-analysis study which has been recently validated by Jinghua et al.^40^. These findings are in contradiction with previous results published by Nilsson et al.^41^ that suggest that downregulation of hsa-miR-92a-3p is associated with aggressive BC features and increased tumour macrophage infiltration. In relation to hsa-miR-320d action in BC, Cava et al.^42^ found that its downregulation favours BC progression. To the best of our knowledge, no other study has investigated the role of hsa-miR-320d in BC progression, therefore it is not possible to compare our result with other data coming from recent literature. According to Maltseva et al.^43^, hsa-miR-193a-5p is less expressed in inflammatory BC patients and is known to play a suppressive role in BC. This statement is in contradiction with the findings in Li et al.^44^ that state that long non-coding RNA small nucleolar RNA host gene 1 (SNG1) activates the HOXA1 expression via sponging hsa-miR-193a-5p in BC progression. Finally, the role of hsa-miR-30b has been shown to be versatile, as a recent review points out^45^. Members of the hsa-miR-30 family play a role in the regulation of tumorigenesis, interference with tumour invasion and metastasis, as well as reversal of drug resistance. Nevertheless, some hsa-miR-30 family members have independent protective effects on the prognosis of BC patients. Surprisingly, among the patients of the AHUS dataset, hsa-miR-200c is oncogenic in 100% of the cases, with single and associative $\beta$ coefficients remaining always positive. Therefore, hsa-miR-200c cannot be qualified as *antagonistic* within our study. However, this finding is in contradiction with previously published research, where hsa-miR-200c is known to be tumor suppressing in BC^46,47^. Song et al. found that hsa-miR-200c inhibits the AKT and ERK pathways by directly targeting KRAS. Repression of KRAS by hsa-miR-200c suppressed the proliferation and survival of BC cells in vitro and in vivo. It is therefore surprising that our results are in contradiction with well-established evidence. In order to understand this paradoxical result, we have drawn the hsa-mir-200c network from the AHUS data set (cf Fig. 8). One can notice that the miRNAs most commonly associated with hsa-miR-200c in our study are hsa-miR-449a (frequency of association with hsa-miR-200c: 100%), hsa-miR-125b-2* (frequency of association: 94%), and hsa-miR-155 (frequency of association: 89 %). These four miRNAs seem therefore to act together, particularly hsa-miR-200c and hsa-miR-449 that form a twin pair. Interestingly, there is no research in the recent literature linking these two miRNAs in BC. The association of hsa-miR-200c and hsa-miR-125b has been studied in a recent work where no significant correlation between these two miRNAs was observed^48^. The hypothesis that we put forward to explain our counter-intuitive result is that in this cohort, the oncogenic function of hsa-miR-200c is stabilized by its high connection with hsa-miR-449a. However this hypothesis needs to be investigated through further research.

**Table 4:** Single (i.e. the estimated value of a $\beta$ coefficient when considering a single miRNA in the logistic model) and associative (i.e. the different values that a miRNA specific $\beta$ coefficient takes in each of the *SWAG* models in which it is present) coefficients (median values and range) for the *antagonistic* miRNAs present in at least 10% of the models.

miRNA	Single $\beta$	Median associative $\beta$	Associative $\beta$ Range
hsa-miR-92a	− 0.736	0.939	(0.095; 1.315)
hsa-miR-320d	− 1.064	1.174	(− 0.412; 2.543)
hsa-miR-193a-5p	− 1.734	1.114	(− 1.318, 1.225)
hsa-miR-30b	0.694	− 0.753	(− 1.422; 0.595)

Associative ranges and not confidence intervals are shown since some of the coefficients display a bi-modal distribution.

### An example of paradigmatic substitution: hsa-miR-140-3p and hsa-miR-30b

Among the 112 selected models, two of them show a perfect discriminating power (cf. Table 1). These two differ only by one miRNA: hsa-miR-140-3p and hsa-miR-30b respectively. From a linguistic point of view, these two miRNAs can be seen as synonyms, meaning that they can be swapped without affecting the meaning of the ”sentence” (the predictive power of the model). As stated previously, miRNAs are short endogenous noncoding RNAs that regulate their target messenger RNAs by promoting their degradation or by repressing their translation^49^. It is therefore intuitive to look at the set of target mRNAs associated with each of these regulatory factors and to determine their intersection. By doing so, we can create a list of common target genes. We acquired information on the targets of these two miRNAs from the http://mirbase.org/ platform^50–54^, and^55^. The targets of these two miRNAs were obtained by crossing information from the TargetScanvert database (http://www.targetscan.org/)^56,57^ and the miRDB database (http://mirdb.org/)^58^. The results are shown in Table 5.

**Table 5:** hsa-miR-140-3p and hsa-miR-30b common targets.

Gene symbol	Gene name
NDST1	N-deacetylase and N-sulfotransferase 1
CCNT2	Cyclin T2
USP49	Ubiquitin specific peptidase 49
ZC3H6	Zinc finger CCCH-type containing 6
DTX4	Deltex E3 ubiquitin ligase 4
CDK6	Cyclin dependent kinase 6
SRGAP3	SLIT-ROBO Rho GTPase activating protein 3
RAB21	RAB21, member RAS oncogene family
P2RY2	Purinergic receptor P2Y2
GLG1	Golgi glycoprotein 1
KCNB1	Potassium voltage-gated channel subfamily B member 1
TNKS	Tankyrase
UBN2	Ubinuclein 2
RFT1	RFT1 homolog
ARID2	AT-rich interaction domain 2
TYRO3	TYRO3 protein tyrosine kinase
EIF5A2	Eukaryotic translation initiation factor 5A2
HPCAL4	Hippocalcin like 4

These targets were selected by crossing information coming from the http://mirdb.org/ and http://www.targetscan.org/ databases.

Among the target genes that are common to hsa-miR-140-3p and hsa-miR-30b, some are well known to play a pivotal role in cancer progression. In this direction, one can cite USP49^59^, CDK6^60^, RAB21^61^, P2RY2^62^, TNKS^63^, ARID2^64^, TYRO3^65^, EIF5A2^66^. This set of common targets may explain why these two miRNAs are ”synonyms” and can be exchanged in predictive models without any harm. The latent functions of these putative target genes is shown in Table 6.

**Table 6:** Function of hsa-miR-140-3p and hsa-miR-30b targets involved in cancer pathophysiology.

Gene symbol	Gene Function
USP49	Histone H2B lysine deubiquitination/mRNA splicing
CDK6	Cell dedifferentiation/cell division
RAB21	Rab protein signal transduction
P2RY2	Cellular ion homeostasis/cellular response to ATP
TNKS	Cell division/mitotic spindle organisation
ARID2	Negative regulation of cell migration and cell population proliferation
TYRO3	Apoptotic cell clearance/cell adhesion and migration
EIF5A2	mRNA transport/regulation of cell population proliferation

Source: https://ensembl.org/ and https://uniprot.org/.

### External validation

In order to provide support to the results presented so far, we performed a validation analysis on a separate dataset collected by the same research team (with the same machines) as the data used for this work. With this choice, we aimed at minimizing the impact of factors such as population selection, batch effect and experimental conditions on our results. We underline that all the figures and tables produced for this analysis are presented in the supplementary material. The validation dataset is made available by Aure et al.^67^ on the Gene Expression Omnibus (GEO) database as a SuperSeries record with accession number GSE58215 at: https://www.ncbi.nlm.nih.gov/geo/query/acc.cgi?acc=GSE58215. miRNA-expression profiling was obtained for 283 patients belonging to the Oslo2 cohort together with their pam50^68^ gene signature classification. In this validation analysis, we first assess the capacity of the SWAG set of 112 models (found on the original AHUS data) to distinguish between normal (25 patients) and other breast cancer types (258 patients). For this purpose, we obtained the SWAG models predictions for the new data (using coefficients taken from the AHUS dataset) and then evaluated the classification performance of these models in terms of accuracy, sensitivity, specificity as well as positive and negative predictive values at the standard logistic cut-off level of 0.5 (cf. S1 Fig. 1). Then, similarly to what was done for the AHUS dataset, we constructed the ROC curve (cf. S1 Fig. 2) of lasso (in red) with the ROC region (in gray) produced by the 112 *SWAG* models on the new dataset. Another goal of this validation analysis, was to support the findings on the *antagonistic* behavior of miRNAs. For this purpose, we fit the same 112 models on the validation dataset to analyse their single and associative effects (coefficients). We present in S1 Table 6 and S1 Table 7 the results of this external validation. These two tables are the validation counterparts of Table 3 and Table 4 respectively. Regarding the overall conclusions of this analysis, we can say that the prediction performance is reasonably preserved (see S1 Figs. 1 and 2) for the set of SWAG models. We also confirm the results obtained in the primary study for three out of four *antagonistic* miRNAs (see the comparison between Table 4 and S1 Table 7). Indeed single and associative coefficients are different for hsa-miR-92a, hsa-miR-320d, hsa-miR-193a-5p thus characterizing them as *antagonistic* in both datasets. Moreover, the signs diverge in the same direction in both analyses: negative for the single coefficient and positive for the associative one. The results for hsa-miR-30b are however different since its single coefficient is positive in the AHUS (primary) dataset while it is negative in the validation one. Nevertheless we consider this discrepancy not surprising because the role of hsa-miR-30b has been shown to be versatile^45^, as already explained in the *oncogenic or protective role of miRNAs* subsection. In a similar way we also confirm the signs of seven of the eight most frequently selected genes (see the comparison between Table 3 and S1 Table 6). On the other hand hsa-miR-155 shows some *antagonistic* behavior given the presence of both positive and negative associative coefficients. To the extent of our analyses, this inconsistency supports the assertion that no definite role can be assigned to this miRNA.

In conclusion, given that a versatile role of miRNAs in BC progression is quite a common finding in recent literature, our results give a statistical basis to this allegation and suggest that the oncogenic or protective role of some mi-RNAs may also depend on the ”network” (or syntagmatic axis) in which they are inserted.

## Discussion

With regard to our research questions, we can firstly conclude that it is possible to differentiate normal breast tissue from breast carcinoma by using miRNAs as biomarkers with reasonable sensitivity and specificity. Secondly, some selected miRNAs behave in opposite ways according to the models in which they are embedded. We decided to call these miRNAs, whose action is conditioned by their insertion in a syntagmatic axis, *antagonistic* micro RNAs. Thirdly, any model selection method such as the one used for this work (*SWAG*) that gives the opportunity to build ”horizontal” and ”vertical” axes can point to latent biological functions and help researchers develop new hypotheses. In our case, regarding hsa-miR-140-3p and hsa-miR-30b, some latent cell functions such as cell division and differentiation, mRNA splicing and transport as well as cellular ion homeostasis appear to be highly relevant.

According to Stepanenko et al.^10^, cancer evolution is a stochastic process both at the genome and gene levels. Most tumors contain multiple genetic subclones, evolving in either succession or in parallel, either in a linear or branching manner, with heterogeneous genome and gene alterations, extensively rewired signaling networks, and addicted to multiple oncogenes easily switching with each other during cancer progression and medical intervention. Hundreds of discovered cancer genes or gene products are classified according to whether they function in an oncogenic or protective manner in a cancer cell. However, there are many cancer “*gene-chameleons*” , which behave in opposite manners in different experimental settings showing what Stepanenko calls “antagonistic duality”. These statements find confirmation in our study. This antagonistic duality affects not only genes, but also miRNAs. For this subgroup, the distribution of the $\beta$ coefficients, either single or associative, include the value zero, thereby indicating an ambiguous or dualistic effect. These results are in line with the most recent literature about their action in BC progression. Indeed, according to Wong et al., genetic variants, many of which fall below statistical significance, can influence disease susceptibility^69^. This finding has prompted theories such as the *omnigenic* model, where any gene expressed in a disease-relevant tissue can affect core disease genes, and thus disease risk, through interactions in a complex interconnected network. Our research fits into this conceptual framework by designing interpretable networks for non-coding miRNAs. Further studies taking into account this versatile effect according to net-like structures are needed.

However, a major concern is how to translate these findings into clinical practice, especially in the context of genetic counselling. In this perspective, solely considering the predictive/diagnostic need, one could rely on statistical or machine learning tools such as model averaging^70^ or ensemble learning^71^ where the predictive/diagnostic power of multiple (possibly contradicting) models is enhanced by combining them in specific ways. More generally though, if some (or perhaps most of) non-coding RNAs exhibit antagonistic duality, the implementation of precision medicine at the patient level may be difficult. As discussed by Nakagawa et al.^72^, due to the diversity of genomes and cancer phenotypes, interpretation of the abundant genomic information from whole-genome sequencing (WGS), especially non-coding and structural variants, requires analysis of large-scale WGS data integrated with RNA-Seq, epigenomic, immunogenomic, and clinico-pathological information. A multi-level atlas of this integrated information may be the next frontier in cancer genomics. In this sense, Stuart et al.^73^ may have pointed in this direction with their comprehensive integration of single cell data.

Our research places itself within the emerging field of artificial intelligence^74^. With the advent of Big Data and the ever-increasing storage and computing power, the challenge has shifted from collecting data to turning it into meaningful and actionable insights. This challenge requires that we leave on the side of the road statistical methods that select genomic items taken in isolation, and that we favour methods that scrutinize biological systems. By producing net-like combinations of equivalent models, it is possible to shed light on the latent biological confounding variables that are usually ignored and may reverse the effect of the considered Omics feature. To conclude, the added value of our research is fourfold: (i) predictive models with high (or optimal) predictive abilities are not unique, but belong to a set of equivalent and, in some sense, exchangeable models; (ii) our results indicate that miRNAs are not isolated items but are integrated in two-dimensional statistical axes. Their function cannot be inferred independently of the other components of the syntagmatic or horizontal axis; (iii) some miRNAs are exchangeable in terms of predictive ability and point to latent biological functions; (iv) conflicting results in the literature suggest that a protective or an oncogenic effect cannot be definitely assigned to any miRNA (even within the same sets of data). Data-driven nets may help biologists in building new hypotheses and experimental designs in order to decipher the function of non-coding RNAs, which may act in antagonistic ways according to the organization in which they are embedded.

## Supplementary Information

Supplementary Information.

## Data Availability

The statistical analysis performed in this study is based on the data presented in Haakensen et al.^6^ available on the open access ArrayExpress platform at: https://www.ebi.ac.uk/arrayexpress/experiments/E-MTAB-3759/?query=AHUS. The validation dataset is made available by Aure et al.^67^ on the Gene Expression Omnibus (GEO) database as a SuperSeries record with accession number GSE58215 at: https://www.ncbi.nlm.nih.gov/geo/query/acc.cgi?acc=GSE58215. To promote reproducibility and replicability, the SWAG is available as an R package on CRAN and at https://github.com/SMAC-Group/SWAG-R-Package/ for its development version. We provide also a public repository at https://github.com/SMAC-Group/swag_breast_cancer where we present all the codes necessary to replicate the findings presented in this article.
