# Supplementary material for: Evidence of antagonistic predictive effects of miRNAs in breast cancer cohorts through data-driven networks
Source: Sci Rep. 2022 Mar 25;12:5166. doi: 10.1038/s41598-022-08737-5 (PMC8956684; doi:10.1038/s41598-022-08737-5)
Supplement: Supplementary file 1 — Supplementary Information. [file 41598_2022_8737_MOESM1_ESM.pdf]

Supplementary Information

Figures

S1 Figure 1. Comparison between Lasso and *SWAG* on the validation dataset [1]. We compare accuracy, sensitivity, specificity, negative predictive value (NPV), positive predictive value (PPV) of the lasso estimates with the ranges (i.e. smallest-to-largest value intervals) of the same metrics for the 112 *SWAG* models. We do not present the related 95% percentile bootstrap confidence intervals for the lasso estimates since they are not informative (i.e. mostly giving $[0,1]$ intervals). All evaluations have been made out-of-sample (i.e. on a balanced validation dataset) at the standard 0.5 cut-off of logistic regression.

	lasso estimates	*SWAG*	
Accuracy	0.56	[0.60,0.87]	
Sensitivity	1	[0.60,0.90]	
Specificity	0.20	[0.52,84]	
NPV	1	[0.68,0.91]	
PPV	0.50	[0.54,0.82]	

S1 Figure 2. ROC curve comparison between Lasso and *SWAG* on the validation dataset [1]. We present the ROC curve of lasso (in red) with the ROC region (in gray) produced by the 112 *SWAG* models. We obtain the ROC region for the set of *SWAG* models considering first all the 112 individual model ROC curves and then coloring in gray the area which enclose all the 112 ROC curves at the same time.

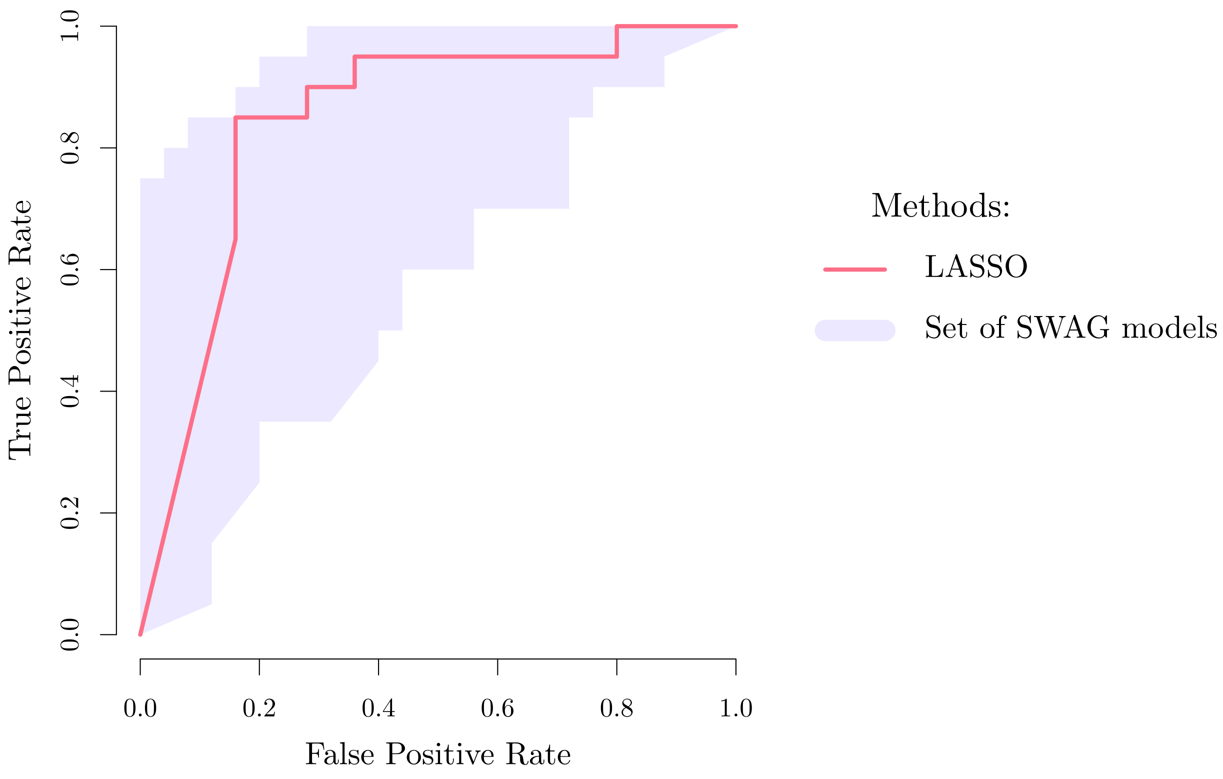

Tables

S1 Table 1. List of all the 45 miRNAs selected by the *SWAG*.

hsa-let-7c	hsa-miR-21	hsa-miR-155	hsa-miR-425
hsa-miR-103	hsa-miR-21*	hsa-miR-181b	hsa-miR-429
hsa-miR-1181	hsa-miR-210	hsa-miR-181d	hsa-miR-449a
hsa-miR-125b-2*	hsa-miR-30b	hsa-miR-183	hsa-miR-486-5p
hsa-miR-1274a	hsa-miR-320c	hsa-miR-193a-5p	hsa-miR-497
hsa-miR-130b	hsa-miR-320d	hsa-miR-193b*	hsa-miR-551b
hsa-miR-139-3p	hsa-miR-328	hsa-miR-196a	hsa-miR-622
hsa-miR-139-5p	hsa-miR-342-3p	hsa-miR-200a	hsa-miR-629*
hsa-miR-140-3p	hsa-miR-342-5p	hsa-miR-200a*	hsa-miR-92a
hsa-miR-141	hsa-miR-375	hsa-miR-200b	hsa-miR-96
hsa-miR-145	hsa-miR-378	hsa-miR-151-3p	hsa-miR-378*
hsa-miR-200c			

S1 Table 2. List of all the models with 4 miRNAs selected by the *SWAG*.

miRNA 1	miRNA 2	miRNA 3	miRNA 4
hsa-let-7c	hsa-miR-125b-2*	hsa-miR-1274a	hsa-miR-21
hsa-miR-1181	hsa-miR-125b-2*	hsa-miR-328	hsa-miR-342-3p
hsa-miR-1274a	hsa-miR-139-3p	hsa-miR-21	hsa-miR-92a
hsa-miR-1274a	hsa-miR-139-3p	hsa-miR-139-5p	hsa-miR-21
hsa-miR-125b-2*	hsa-miR-155	hsa-miR-200c	hsa-miR-449a
hsa-miR-1274a	hsa-miR-139-3p	hsa-miR-193a-5p	hsa-miR-21
hsa-miR-1274a	hsa-miR-181b	hsa-miR-21	hsa-miR-21*
hsa-miR-1274a	hsa-miR-21	hsa-miR-30b	hsa-miR-92a
hsa-miR-1274a	hsa-miR-139-3p	hsa-miR-21	hsa-miR-378
hsa-miR-1274a	hsa-miR-21	hsa-miR-320d	hsa-miR-429
hsa-miR-125b-2*	hsa-miR-196a	hsa-miR-21	hsa-miR-486-5p
hsa-miR-1274a	hsa-miR-139-3p	hsa-miR-21	hsa-miR-551b
hsa-miR-1274a	hsa-miR-21	hsa-miR-320d	hsa-miR-96

S1 Table 3. List of all the models with 5 miRNAs selected by the *SWAG*. (Part 1)

miRNA 1	miRNA 2	miRNA 3	miRNA 4	miRNA 5
hsa-let-7c	hsa-miR-125b-2*	hsa-miR-1274a	hsa-miR-139-3p	hsa-miR-21
hsa-let-7c	hsa-miR-125b-2*	hsa-miR-1274a	hsa-miR-155	hsa-miR-21
hsa-let-7c	hsa-miR-125b-2*	hsa-miR-1274a	hsa-miR-21	hsa-miR-320d
hsa-let-7c	hsa-miR-125b-2*	hsa-miR-1274a	hsa-miR-21	hsa-miR-486-5p
hsa-let-7c	hsa-miR-125b-2*	hsa-miR-1274a	hsa-miR-193a-5p	hsa-miR-21
hsa-let-7c	hsa-miR-125b-2*	hsa-miR-1274a	hsa-miR-193b*	hsa-miR-21
hsa-let-7c	hsa-miR-125b-2*	hsa-miR-1274a	hsa-miR-21	hsa-miR-378
hsa-let-7c	hsa-miR-125b-2*	hsa-miR-1274a	hsa-miR-21	hsa-miR-328
hsa-let-7c	hsa-miR-125b-2*	hsa-miR-1274a	hsa-miR-21	hsa-miR-429
hsa-let-7c	hsa-miR-125b-2*	hsa-miR-1274a	hsa-miR-21	hsa-miR-622
hsa-miR-103	hsa-miR-1274a	hsa-miR-139-3p	hsa-miR-21	hsa-miR-30b
hsa-miR-103	hsa-miR-125b-2*	hsa-miR-1274a	hsa-miR-21	hsa-miR-497
hsa-miR-125b-2*	hsa-miR-1274a	hsa-miR-21	hsa-miR-320c	hsa-miR-320d
hsa-miR-125b-2*	hsa-miR-1274a	hsa-miR-21	hsa-miR-328	hsa-miR-92a
hsa-miR-103	hsa-miR-125b-2*	hsa-miR-1274a	hsa-miR-328	hsa-miR-342-3p
hsa-miR-1274a	hsa-miR-130b	hsa-miR-139-3p	hsa-miR-181b	hsa-miR-21
hsa-miR-1274a	hsa-miR-130b	hsa-miR-181b	hsa-miR-21	hsa-miR-21*
hsa-miR-1181	hsa-miR-125b-2*	hsa-miR-139-3p	hsa-miR-328	hsa-miR-342-3p
hsa-miR-125b-2*	hsa-miR-130b	hsa-miR-139-3p	hsa-miR-200c	hsa-miR-449a
hsa-miR-1274a	hsa-miR-139-3p	hsa-miR-139-5p	hsa-miR-21	hsa-miR-320d
hsa-miR-1274a	hsa-miR-139-3p	hsa-miR-140-3p	hsa-miR-21	hsa-miR-92a
hsa-miR-1274a	hsa-miR-139-3p	hsa-miR-151-3p	hsa-miR-21	hsa-miR-92a
hsa-miR-125b-2*	hsa-miR-139-3p	hsa-miR-155	hsa-miR-200c	hsa-miR-449a
hsa-miR-1274a	hsa-miR-139-3p	hsa-miR-181b	hsa-miR-21	hsa-miR-449a
hsa-miR-1274a	hsa-miR-139-3p	hsa-miR-181b	hsa-miR-183	hsa-miR-21
hsa-miR-1274a	hsa-miR-139-3p	hsa-miR-181b	hsa-miR-21	hsa-miR-320d
hsa-miR-1274a	hsa-miR-139-3p	hsa-miR-181d	hsa-miR-21	hsa-miR-92a
hsa-miR-1274a	hsa-miR-139-3p	hsa-miR-200a	hsa-miR-21	hsa-miR-92a
hsa-miR-1274a	hsa-miR-139-3p	hsa-miR-21	hsa-miR-320c	hsa-miR-320d
hsa-miR-1274a	hsa-miR-139-3p	hsa-miR-21	hsa-miR-320d	hsa-miR-92a
hsa-miR-1274a	hsa-miR-139-3p	hsa-miR-21	hsa-miR-342-3p	hsa-miR-92a
hsa-miR-1274a	hsa-miR-139-3p	hsa-miR-21	hsa-miR-449a	hsa-miR-92a
hsa-miR-1274a	hsa-miR-139-3p	hsa-miR-21	hsa-miR-92a	hsa-miR-96
hsa-miR-1181	hsa-miR-125b-2*	hsa-miR-139-5p	hsa-miR-328	hsa-miR-342-3p
hsa-miR-1274a	hsa-miR-139-3p	hsa-miR-139-5p	hsa-miR-21	hsa-miR-449a
hsa-miR-1274a	hsa-miR-139-3p	hsa-miR-139-5p	hsa-miR-183	hsa-miR-21
hsa-miR-1274a	hsa-miR-139-3p	hsa-miR-139-5p	hsa-miR-21	hsa-miR-92a

S1 Table 4. List of all the models with 5 miRNAs selected by the *SWAG*. (Part 2)

miRNA 1	miRNA 2	miRNA 3	miRNA 4	miRNA 5
hsa-miR-125b-2*	hsa-miR-139-5p	hsa-miR-155	hsa-miR-200c	hsa-miR-449a
hsa-miR-1274a	hsa-miR-139-5p	hsa-miR-21	hsa-miR-30b	hsa-miR-92a
hsa-miR-1274a	hsa-miR-140-3p	hsa-miR-21	hsa-miR-30b	hsa-miR-92a
hsa-miR-1274a	hsa-miR-140-3p	hsa-miR-21	hsa-miR-328	hsa-miR-92a
hsa-miR-1274a	hsa-miR-139-3p	hsa-miR-141	hsa-miR-181b	hsa-miR-21
hsa-miR-1274a	hsa-miR-139-3p	hsa-miR-141	hsa-miR-21	hsa-miR-92a
hsa-miR-125b-2*	hsa-miR-145	hsa-miR-155	hsa-miR-200c	hsa-miR-449a
hsa-miR-1274a	hsa-miR-139-3p	hsa-miR-155	hsa-miR-196a	hsa-miR-21
hsa-miR-1274a	hsa-miR-139-3p	hsa-miR-155	hsa-miR-21	hsa-miR-375
hsa-miR-1274a	hsa-miR-139-3p	hsa-miR-155	hsa-miR-193a-5p	hsa-miR-21
hsa-miR-1274a	hsa-miR-139-3p	hsa-miR-155	hsa-miR-21	hsa-miR-378
hsa-miR-1274a	hsa-miR-139-3p	hsa-miR-181b	hsa-miR-181d	hsa-miR-21
hsa-miR-1274a	hsa-miR-181b	hsa-miR-21	hsa-miR-21*	hsa-miR-92a
hsa-miR-1274a	hsa-miR-139-3p	hsa-miR-181b	hsa-miR-21	hsa-miR-342-3p
hsa-miR-1274a	hsa-miR-139-3p	hsa-miR-181b	hsa-miR-21	hsa-miR-425
hsa-miR-1274a	hsa-miR-139-3p	hsa-miR-181b	hsa-miR-21	hsa-miR-96
hsa-miR-1274a	hsa-miR-139-3p	hsa-miR-181d	hsa-miR-193a-5p	hsa-miR-21
hsa-miR-1274a	hsa-miR-139-3p	hsa-miR-193a-5p	hsa-miR-21	hsa-miR-449a
hsa-miR-1274a	hsa-miR-139-3p	hsa-miR-193a-5p	hsa-miR-196a	hsa-miR-21
hsa-miR-1274a	hsa-miR-139-3p	hsa-miR-193a-5p	hsa-miR-200a*	hsa-miR-21
hsa-miR-1274a	hsa-miR-193a-5p	hsa-miR-21	hsa-miR-30b	hsa-miR-92a
hsa-miR-1274a	hsa-miR-193a-5p	hsa-miR-21	hsa-miR-320c	hsa-miR-320d
hsa-miR-1274a	hsa-miR-139-3p	hsa-miR-193a-5p	hsa-miR-21	hsa-miR-378
hsa-miR-1274a	hsa-miR-139-3p	hsa-miR-193a-5p	hsa-miR-21	hsa-miR-551b
hsa-miR-1274a	hsa-miR-139-3p	hsa-miR-193a-5p	hsa-miR-21	hsa-miR-96
hsa-miR-125b-2*	hsa-miR-155	hsa-miR-193b*	hsa-miR-200c	hsa-miR-449a
hsa-miR-1274a	hsa-miR-139-3p	hsa-miR-196a	hsa-miR-21	hsa-miR-92a
hsa-miR-1274a	hsa-miR-140-3p	hsa-miR-181b	hsa-miR-196a	hsa-miR-21
hsa-miR-125b-2*	hsa-miR-155	hsa-miR-200a	hsa-miR-200c	hsa-miR-449a
hsa-miR-1274a	hsa-miR-200a	hsa-miR-21	hsa-miR-30b	hsa-miR-92a
hsa-miR-125b-2*	hsa-miR-155	hsa-miR-200a*	hsa-miR-200c	hsa-miR-449a
hsa-miR-125b-2*	hsa-miR-130b	hsa-miR-200a*	hsa-miR-200b	hsa-miR-449a
hsa-miR-125b-2*	hsa-miR-155	hsa-miR-200b	hsa-miR-200c	hsa-miR-449a
hsa-miR-1274a	hsa-miR-130b	hsa-miR-21	hsa-miR-21*	hsa-miR-320d
hsa-miR-1274a	hsa-miR-21	hsa-miR-21*	hsa-miR-320c	hsa-miR-320d
hsa-miR-1274a	hsa-miR-139-3p	hsa-miR-181b	hsa-miR-193a-5p	hsa-miR-210
hsa-miR-1274a	hsa-miR-139-3p	hsa-miR-181b	hsa-miR-21	hsa-miR-210

S1 Table 5. List of all the models with 5 miRNAs selected by the *SWAG*. (Part 3)

miRNA 1	miRNA 2	miRNA 3	miRNA 4	miRNA 5
hsa-miR-125b-2*	hsa-miR-155	hsa-miR-200c	hsa-miR-30b	hsa-miR-449a
hsa-miR-1274a	hsa-miR-21	hsa-miR-30b	hsa-miR-328	hsa-miR-92a
hsa-miR-1274a	hsa-miR-21	hsa-miR-30b	hsa-miR-378	hsa-miR-92a
hsa-miR-125b-2*	hsa-miR-1274a	hsa-miR-21	hsa-miR-320c	hsa-miR-92a
hsa-miR-1274a	hsa-miR-139-3p	hsa-miR-21	hsa-miR-320c	hsa-miR-92a
hsa-miR-1181	hsa-miR-125b-2*	hsa-miR-320d	hsa-miR-328	hsa-miR-342-3p
hsa-miR-125b-2*	hsa-miR-130b	hsa-miR-200c	hsa-miR-328	hsa-miR-449a
hsa-miR-1274a	hsa-miR-21	hsa-miR-320c	hsa-miR-320d	hsa-miR-328
hsa-miR-125b-2*	hsa-miR-155	hsa-miR-200c	hsa-miR-342-5p	hsa-miR-449a
hsa-miR-1274a	hsa-miR-139-3p	hsa-miR-21	hsa-miR-378	hsa-miR-449a
hsa-miR-125b-2*	hsa-miR-155	hsa-miR-200c	hsa-miR-378	hsa-miR-449a
hsa-miR-1181	hsa-miR-125b-2*	hsa-miR-328	hsa-miR-342-3p	hsa-miR-378*
hsa-miR-125b-2*	hsa-miR-155	hsa-miR-200c	hsa-miR-378*	hsa-miR-449a
hsa-miR-1274a	hsa-miR-21	hsa-miR-30b	hsa-miR-378*	hsa-miR-92a
hsa-miR-125b-2*	hsa-miR-155	hsa-miR-200c	hsa-miR-429	hsa-miR-449a
hsa-miR-139-3p	hsa-miR-155	hsa-miR-200c	hsa-miR-449a	hsa-miR-497
hsa-miR-1274a	hsa-miR-21	hsa-miR-320c	hsa-miR-320d	hsa-miR-449a
hsa-miR-1274a	hsa-miR-21	hsa-miR-30b	hsa-miR-486-5p	hsa-miR-92a
hsa-miR-125b-2*	hsa-miR-155	hsa-miR-200c	hsa-miR-449a	hsa-miR-497
hsa-miR-1181	hsa-miR-125b-2*	hsa-miR-328	hsa-miR-342-3p	hsa-miR-551b
hsa-miR-125b-2*	hsa-miR-155	hsa-miR-200c	hsa-miR-449a	hsa-miR-551b
hsa-miR-1274a	hsa-miR-21	hsa-miR-30b	hsa-miR-551b	hsa-miR-92a
hsa-miR-1181	hsa-miR-125b-2*	hsa-miR-328	hsa-miR-342-3p	hsa-miR-629*
hsa-miR-1181	hsa-miR-125b-2*	hsa-miR-328	hsa-miR-342-3p	hsa-miR-92a
hsa-miR-103	hsa-miR-125b-2*	hsa-miR-1274a	hsa-miR-21	hsa-miR-96

S1 Table 6. We present the single (i.e. the estimated value of a β coefficient when considering a single miRNA in the logistic model) and associative (i.e. the different values that a miRNA specific β coefficient takes in each of the *SWAG* models in which it is present) coefficients (median values and range) obtained on the validation dataset [1] for the eight most frequently selected miRNAs of the AHUS dataset [2] (primary study).

miRNA	Single β	Median associative β	Associative β Range
**hsa miR-1274a**	0.330	0.200	(0.017; 0.668)
**hsa−miR−21**	1.190	1.130	(0.602; 1.512)
**hsa miR-139-3p**	-1.020	-0.695	(-1.178, -0.110)
**hsa-miR-125b-2***	-1.840	-1.358	(-2.223; -0.482)
**hsa−miR−92a**	-0.260	0.068	(-0.472; 0.623)
**hsa−miR−449a**	0.470	0.492	(0.379; 0.598)
hsa−miR−155	0.360	0.489	(-0.147; 0.606)
**hsa miR-200c**	0.820	0.747	(0.580; 0.891)

We present in bold the miRNAs whose single and associative β signs are confirmed in both the AHUS (primary) and validation dataset.

S1 Table 7. We present the single (i.e. the estimated value of a β coefficient when considering a single miRNA in the logistic model) and associative (i.e. the different values that a miRNA specific β coefficient takes in each of the *SWAG* models in which it is present) coefficients (median values and range) obtained on the validation dataset [1] for the *antagonistic* miRNAs present in at least 10% of the models of the AHUS dataset [2] (primary study).

miRNA	Single β	Median associative β	Associative β Range
**hsa-miR-92a**	-0.260	0.068	(-0.472; 0.623)
**hsa-miR-320d**	-0.130	0.305	(-0.518; 0.627)
**hsa-miR-193a-5p**	-0.270	0.052	(-0.413, 0.188)
hsa-miR-30b	-0.170	-0.291	(-0.664; -0.052)

We present in bold the miRNAs whose *antagonistic* role is confirmed in both the AHUS (primary) and validation dataset.

References

1. Aure MR, Jernström S, Krohn M, Vollan HKM, Due EU, Rødland E, et al. Integrated analysis reveals microRNA networks coordinately expressed with key proteins in breast cancer. Genome medicine. 2015;7(1):1–17.

2. Haakensen VD, Nygaard V, Greger L, Aure MR, Fromm B, Bukholm IR, et al. Subtype-specific micro-RNA expression signatures in breast cancer progression. International journal of cancer. 2016;139(5):1117–1128.
